# Neuronal antibodies in patients with suspected or confirmed sporadic Creutzfeldt-Jakob disease

**DOI:** 10.1136/jnnp-2014-308695

**Published:** 2014-09-22

**Authors:** Meghan Rossi, Simon Mead, John Collinge, Peter Rudge, Angela Vincent

**Affiliations:** 1 Nuffield Department of Clinical Neurosciences, University of Oxford, Oxford, UK; 2 NHS National Prion Clinic, National Hospital for Neurology and Neurosurgery, University College London Hospitals NHS Trust, London, UK; 3 MRC Prion Unit, Department of Neurodegenerative Disease, UCL Institute of Neurology, London, UK

**Keywords:** PRION, NMDA, NEUROIMMUNOLOGY, IMMUNOLOGY, LIMBIC SYSTEM

## Abstract

**Objectives:**

There have been reports of patients with antibodies to neuronal antigens misdiagnosed as sporadic Creutzfeldt-Jakob disease (sCJD). Conversely, low levels of antibodies to neuronal proteins have been reported in patients with sCJD. However, the frequency of misdiagnoses, or of antibodies in patients with subsequently confirmed sCJD, is not clear.

**Methods:**

We reviewed 256 consecutive cases of sCJD seen in the National Prion Clinic, of whom 150 had sera previously referred for selected antibody tests. Eighty-two available samples were retested for antibodies to *N*-methyl-d-aspartate receptor (NMDAR), the glycine receptor (GlyR), voltage-gated potassium channel (VGKC)-complex and the associated proteins, leucine-rich glioma inactivated 1 (LGI1) and contactin-associated protein 2 (CASPR2).

**Results:**

Four of the initial 150 sera referred were positive; two had antibodies to NMDAR, and two to the VGKC-complex, one of which was also positive for GlyR antibodies. Of the 82 sCJD sera retested, one had VGKC-complex antibodies confirming the previous result, two had CASPR2 and GlyR antibodies and one had CASPR2 and NMDAR antibodies; all antibodies were at low levels. Over the same period three patients with autoimmune encephalitis and high VGKC-complex antibodies were initially referred as sCJD.

**Conclusions:**

This study indicates that <5% patients with sCJD develop serum antibodies to these neuronal antigens and, when positive, only at low titres. By contrast, three patients referred with possible prion disease had a clinical picture in keeping with autoimmune encephalitis and very high VGKC-complex/LGI1 antibodies. Low titres of neuronal antibodies occur only rarely in suspected patients with sCJD and when present should be interpreted with caution.

## Introduction

Autoantibodies to specific neuronal proteins are associated with encephalopathies^1^ ^2^ but these can share clinical features, such as cognitive decline, personality changes and movement disorders, with Creutzfeldt-Jakob disease (CJD).^3^ There have been several case reports and two studies^3^ ^4^ that included patients whose diagnosis of immunotherapy-responsive limbic encephalitis was delayed because of a suspected diagnosis of CJD. Conversely, there have been occasional reports of patients presenting with encephalopathy and low levels of serum antibodies to neuronal protein such as the *N*-methyl-d-aspartate receptor (NMDAR), voltage-gated potassium channel complex (VGKC-complex) or glycine receptor (GlyR), who were later confirmed to have sporadic CJD (sCJD).^5–7^

Although the absence of NMDAR antibodies in 346 referred cerebrospinal fluid (CSF) samples, including samples from 49 confirmed sCJD cases^4^ was reported recently, the frequency of disease-relevant serum antibodies in patients with sCJD prior to diagnosis, and how frequently an incorrect CJD diagnosis could have been averted, have not been studied systematically. Here we report antibody testing in patients seen in the National Prion Clinic, to which all cases of suspected prion disease in the UK are referred. We determined the number of samples sent for antibody testing prior to referral, and then tested or retested all available sera for the most relevant antibodies. Our results indicate that antibodies detected in patients with subsequently-confirmed sCJD are rare and only present at low levels that may not be clinically relevant. We contrast these cases with three patients examined during the same period whose eventual diagnosis was definite autoimmune encephalitis, supported by high titres of VGKC-complex/LGI1 antibodies.

## Methods

Since 2004 all patients in the UK with suspected CJD have been referred jointly to the National Prion Clinic in London and to the National CJD Research and Surveillance Unit in Edinburgh. From 2008, a subset of these patients was recruited into the National Prion Monitoring Cohort, a study designed to determine the natural history of all types of CJD. By June 2013, a total of 486 patients were documented. A total of 456 of these patients were considered to have clinically probable or definite CJD^8^ comprising 256 with sCJD, 9 with variant CJD, 12 with iatrogenic CJD due to treatment with contaminated human pituitary-derived growth hormone and 167 symptomatic or at-risk of inherited prion disease. The remainder had a variety of other, mainly neurodegenerative, conditions, including three with autoimmune encephalitis.

Review of the 256 cases of probable or definite patients with sCJD identified 150 patients for whom serum had been sent to the Clinical Neuroimmunology service in Oxford for a variety of individual antibody assays. No CSF samples had been sent. After compiling the results of all diagnostic tests requested, we retrieved the 82 sera still available in order to screen or rescreen for neuronal antibodies. Antibodies to NMDAR, GlyR, LGI1 and CASPR2 were detected by demonstrating antibody-binding to human embryonic kidney cells transfected with complementary DNA encoding the different antigens, as used in the diagnostic service.^9–14^ VGKC-complex antibodies were determined by immunoprecipitation of ^125^I-α-dendrotoxin-labelled rabbit whole brain extract as also used for diagnosis.^13^ All results were assessed independently by two observers, and positive results were repeated at different dilutions when sufficient sample was available.

## Results

### Referred samples

Neurologists around the UK had requested a total of 305 diagnostic antibody tests for 150 of the 256 patients with sCJD (59%) before referral to the National Prion Clinic. The most commonly requested tests were for VGKC-complex, NMDAR and paraneoplastic antibodies (table 1). Of the 150 sera, two were reported as low positive for NMDAR antibodies and two were positive or low positive for VGKC-complex antibodies. The latter patient was also low positive, then positive, for GlyR antibodies.^7^ The results and normal ranges are given in the table 1. The remaining 146 sera were negative for all requested tests, including antibodies against paraneoplastic antigens, the P/Q-type voltage-gated calcium channel (VGCC) and NMDAR (see table 1).

**Table 1:** Antibodies found in patients with sCJD or limbic encephalitis

150 Sera referred pre-sCJD diagnosis (n=number tests requested)	Positive (% tested) Antibody scores or titres*
VGKC-complex (n=119)	2 (1.7%); 210 pM, 113 pM
NMDAR (n=77)	2 (2.6%); both low positive at 1:20
Paraneoplastic (n=51)	0
VGCC (n=25)	0
GAD (n=16)	0
GlyR (n=6)	1; low positive initially, rising to positive at 1:20; Also VGKC-complex 210 pM
Ganglioside (n=5)	0
MuSK (n=3)	0
AQP4 (n=2)	0
MOG (n=1)	0
MAG (n=1)	0
Total positive sera	4/150 (2.7%)
82 sCJD Sera available for retrospective analysis†	
VGKC-complex	1 (1.2%), confirmed previous requested result
NMDAR	1 (1.2%) low positive at 1:20
GlyR	2 (2.4%), positive at 1:20; and positive 1:100
CASPR2	3 (3.6%) low positive at 1:100, positive at 1:200 and 1:400; each positive for NMDAR (n=1) or GlyR (n=2) antibodies as above
LGI1	0
Total positive sera	4/82 (4.9%)
Referred after admission to the National Prion Clinic as probable sCJD. Final diagnosis limbic encephalitis	
VGKC-complex	3 (3.6%) >5000 pM, two LGI-Ab positive (titres not determined), one untested. One female given immunotherapy recovered, two untreated males died

*Details of the patients with positive antibody tests are included in the online supplementary tables. The screening assays were performed as for all routine samples at 1:20 (NMDAR, GlyR) or 1:100 (CASPR2) and the reports based on visual binding scores of 0 (negative), 1–4 (positive with increasing intensity). Low positive at 1:20 (or 1:100 for CASPR2) infers a score of 1.5; positive infers >1.5. Titres are based on further dilutions of serum until the endpoint dilution which gives a score of 1. Normal values based on healthy and disease controls are <1:20 for NMDAR, LGI1 and GlyR and <1:100 for CASPR2.^12–14^

†All sCJD sera available; postmortem-confirmed n=42; postmortem not performed n=40.

AQP4, aquaporin-4; CASPR2, contactin-associated protein 2; GAD, glutamic acid decarboxylase; GlyR, glycine receptor; LGI1, leucine-rich glioma inactivated 1; MAG, myelin-associated glycoprotein; MOG, myelin oligodendrocyte glycoprotein; NMDAR, *N*-methyl-d-aspartate receptor; sCJD, sporadic Creutzfeldt-Jakob disease; VGCC, voltage-gated calcium channel; VGKC, voltage-gated potassium channel complex.

### Retrieved samples

Sera from 82 of the patients were still available and were systematically tested for serum antibodies to NMDAR, GlyR, VGKC-complexes and the associated proteins LGI1 and CASPR2. Four patients (5%) tested positive. One had VGKC-complex antibodies (confirming the previous requested test). Three had CASPR2 antibodies, two of these were also positive for GlyR antibodies, and one also had NMDAR antibodies. LGI1 antibodies were not found in any sera. The results are detailed in table 1.

### All patients with sCJD with positive antibodies

Between the referred samples and retrieved samples, there were seven patients with sCJD with one or more antibodies detected (<5%, table 1). Details of the patients’ clinical presentations and investigations are given in online supplementary table S1. Their mean age was 68 years and the mean duration was 203 (59–401) days compared to a mean age of 67 years and mean duration of 242 (27–2387) days in the 249 patients with sCJD for whom no antibodies were detected.

### Samples referred from patients with encephalitis

Over the same period of time three additional patients, referred to the National Prion Clinic with a provisional diagnosis of sCJD, were considered more likely to have autoimmune encephalitis. Each had high VGKC-complex antibody titres when tested (>5000 pM, table 1) and two had demonstrated specificity for LGI1. The clinical details are given in online supplementary table S2. One female was treated successfully with a rapid fall in VGKC-complex antibodies and made a complete recovery, but the two males died within a month of testing without treatment.

### Postmortem results

Of the 249 patients who died, 139 had postmortem brain examinations. The diagnosis of CJD was confirmed in all. The diagnosis of encephalitis was confirmed in the one patient with high titres of VGKC-complex antibodies who had an autopsy.

## Discussion

There can be diagnostic confusion at the onset of sCJD. One important immunotherapy-responsive disease that needs to be excluded is VGKC-complex antibody positive limbic encephalitis.^3^ ^4^ Other immune-mediated diseases may be suspected depending on the clinical features. A high proportion of patients with eventual sCJD diagnosis (150/256; 59%) had sera referred for specific antibody assays, the requested tests presumably reflecting their clinical features at presentation. The number of positive results, however, was low, with NMDAR or VGKC-complex antibodies present in only four patients (<5%) and no other antibodies detected at that time. When systematically testing all 82 available sera for NMDAR, GlyR and VGKC-complex antibodies, only 4 (3 additional) were positive (5%) and mostly at relatively low levels; but, during the same period of time, three other patients were identified with high VGKC-complex antibodies (>400 pM). Although two of these died before treatment could be initiated, the remaining patient recovered with immunotherapy. We can therefore conclude that while VGKC-complex or NMDAR antibodies are not a common feature of sCJD, they can be present in rare cases at levels unlikely to be of clinical relevance, as indicted by other studies.^9^^–^12 Higher levels of these antibodies, by contrast, are very likely to be associated with an alternative autoimmune diagnosis. It remains important, therefore, to consider autoimmune encephalitis in the differential diagnosis and to test for the relevant antibodies.

Reassuringly, a high proportion of patients were investigated for possible paraneoplastic or autoimmune forms of encephalitis during their presentation. In spite of this, high levels of VGKC-complex antibodies in two patients were not detected early enough during disease progression. It is important for clinicians to be aware of clinical features that are atypical in sCJD presentation (see online supplementary table S2), such as faciobrachial seizures and autonomic dysfunction, which may indicate an autoimmune disorder. This is particularly relevant if the MRI does not show the typical features of prion disease on diffusion sequences. Furthermore, too much reliance on non-specific markers of degeneration in CSF such as 14.3.3 and S100B proteins, positive in both tested encephalitis patients (see online supplementary table S2), can result in an incorrect diagnosis. However, a high positive VGKC-complex Ab (LGI1-Ab negative) was recently identified in a patient with a new Gerstmann-Straüssler-Scheinker mutation who did not respond to immunotherapies and subsequently died.^15^

None of the patients had CSF referred for testing, likely because the Oxford assays do not require CSF for diagnostic testing and also due to the perceived potential risk with CSF from possible sCJD cases. The recent study of 300 CSF samples, including 49 from patients with definite sCJD from the Barcelona Centre^4^ found that all sCJD samples were negative for NMDAR antibodies. However, given that concentrations in the CSF are lower than that of serum, they could be difficult to detect in patients with the low concentrations reported here.

The reason for the occasional presence of serum antibodies at low levels in sCJD is unclear. It is possible that they occur as a result of extensive and rapid neuronal destruction, as suggested by a case of sCJD where both VGKC-complex and glycine receptor antibodies were identified (case 1, see supplementary table S1).^7^ In this case improvement after immunotherapy was apparent, though not sustained. Some improvement in cognitive function was also observed in case 3 after the patient underwent a trial of immunotherapy (see supplementary table S1). These cases raise the possibility that the autoantibodies identified in patients with sCJD, although unlikely to be primarily pathogenic in patients with a neurodegenerative disorder, may sometimes contribute to the clinical manifestations during the disease process. Further studies on autoantibody levels during the course of sporadic and genetic forms of prion disease, and whether the antibodies contribute to the disease pathophysiology, could provide insight into the complex role of autoantibodies in neurodegenerative diseases.

## Supplementary Material

Web supplement
