# Supplementary material for: Neuronal antibodies in patients with suspected or confirmed sporadic Creutzfeldt-Jakob disease
Source: J Neurol Neurosurg Psychiatry. 2014 Sep 22;86(6):692–4. doi: 10.1136/jnnp-2014-308695 (PMC4453627; doi:10.1136/jnnp-2014-308695)
Supplement: Web supplement [file jnnp-2014-308695-s1.pdf]

Neuronal antibodies in patients with suspected or confirmed sporadic Creutzfeldt-Jakob Disease

Meghan Rossi[1], Simon Mead[2,3], John Collinge[2,3], Peter Rudge[2,3], Angela Vincent[1]

[1]Nuffield Department of Clinical Neurosciences, University of Oxford, Oxford, UK; [2]NHS National Prion Clinic, National Hospital for Neurology and Neurosurgery, University College London Hospitals NHS Trust, London, UK; [3]MRC Prion Unit, Department of Neurodegenerative Diseases, UCL Institute of Neurology, London, UK

Supplementary Table 1.

Probable and definite cases of sCJD with serum antibodies

Patient	Sex, Age	Symptoms at onset	Antibodies originally requested	MRI, EEG,<br>CSF<br>Prion protein gene codon 129**	Antibodies positive	Immunotherapy and response	Final diagnosis
**Case 1**	**M, 68**	Two-year history of insomnia, with irritability and weight loss, muscle spasms in legs, burning feet, developed startle, poor frontal function, pout reflex, increased tone, myoclonus, and myokymia.	Para-neoplastic, NMDAR, GlyR, VGKC	MRI mild frontal atrophy at first.<br>EEG normal and then progressive slowing and delta rhythm.<br>CSF acellular, 14.3.3 positive and S100B raised.<br>VV	Pre-diagnosis VGKC-complex 210 pM; GlyR 1:20 initially rising	IVIG, cyclophosphamide,prednisolone, and plasmapheresis. Slight improvement initially, was not sustained.	Died. Post-mortem (PM) confirmed sCJD. Previously reported[7]

**Case 2**	F, 72	Presented with visual and speech disturbance, gait problems, tremor. On admission, confused, not obeying commands, rigidity of limbs, myoclonus and choreiform movements.	VGKC, NMDAR	MRI Cortical ribboning and restricted diffusion in basal ganglia.<br>EEG slow with periodic complexes.<br>CSF 14.3.3 positive and S100B raised.<br>MM	Pre-diagnosis VGKC 113 pM.	None	Died eight weeks after first symptoms. Presumed sCJD<br>No PM.
**Case 3**	M, 57	Sweating, dystonia, memory impairment, ataxia, urinary retention, paralytic ileus and atrial fibrillation. Mute on admission.	VGKC, NMDAR, MuSK, GlyR	MRI Restricted diffusion caudate, putamen, thalamus with cortical ribboning.<br>EEG diffuse slowing.<br>CSF 14.3.3 positive and S100B raised.	Pre-diagnosis NMDAR 1:20 low positive*	IvMP and IvIG, plasmapheresis. Autonomic and cognitive features improved, but deteriorated subsequently	Died 5 months after first symptoms.<br>PM confirmed CJD.

				MM			
**Case 4**	F, 71	11-month history of withdrawal, decline in speech and navigating, progressing to severe memory problems and hallucinations. Myoclonus and apraxia. On admission, mute, rigidity and myoclonus.	VGKC, NMDAR	MRI restricted diffusion in the cortex.<br><br>EEG slow with triphasic complexes.<br><br>CSF 14.3.3 positive and S100B raised<br><br>MV	Pre-diagnosis NMDAR 1:20 low positive	None	Died 15 months after first symptoms (4 months after admission).<br><br>PM confirmed CJD.
**Case 5**	F, 75	10 weeks of left sided sensory loss, unsteadiness and personality change. Bedbound and incontinent within two months. On admission, disorientation, poor concentration, and severe constructional apraxia. Alien left side. Rigidity and	VGKC, GAD	MRI restricted diffusion in cortical ribbon, especially on right, and basal ganglia.<br><br>EEG generalised slow waves.<br><br>CSF 14.33 positive and S100B raised.	Retrospective CASPR2 1:400 GlyR 1:50	None	Died 3 months from first symptom.<br><br>PM confirmed CJD.

		myoclonus.		MM.			
**Case 6**	M, 70	10 month personality change, insomnia. 5 month cognitive decline, decreasing mobility,hallucinations, rigitdity then akinetic mute.	VGKC, NMDAR	MRI restricted diffusion basal ganglia and thalamus.<br>EEG slow waves.<br>CSF acellular 14.3.3 positive and S100B raised.<br>VV	Retrospective CASPR2 1:100 GlyR 1:100*	None	Died 10 months after first symptoms.<br>PM confirmed CJD.
**Case 7**	M, 55	7 weeks of personality change, confusion, alien limb, loss of speech, visual hallucinations, myoclonus and gait difficulties. Mute, vertical supranuclear gaze paresis, increased tone, severe ataxia and incontinence.	Para-neoplastic (Hu, Yo, Ri etc)	MRI restricted diffusion in cortical ribbon.<br>CSF 14.3.3 positive and S100B raised.<br>EEG PLEDS<br>No prion protein gene sequencing.	Retrospective CASRP2 1:200* NMDAR 1:100*	None	Died one week after admission, two months after first symptoms.<br>Presumed CJD<br>PM refused.

7. Angus-Leppan H, Rudge P, Mead S, et al. Autoantibodies in sporadic Creutzfeldt-Jakob disease. *JAMA Neurol* 2013;70(7):919-22.

The screening assays were performed as for all routine samples at 1:20 (NMDAR, LGI1, GlyR) or 1:100 (CASPR2) and the reports based on visual binding scores of 0 (negative), 1– 4 (positive with increasing intensity). Low positive at 1:20 (or 1:100 for CASPR2) infers a score of 1.5; positive infers >1.5. Titres are based on further dilutions of serum at which the intensity gives a score of 1. Normal values based on healthy and disease controls are <1:20 for NMDAR, LGI1 and GlyR, and <1:100 for CASPR2. [12, 13, 14]

Supplementary Table 2.

Patients with VGKC-complex antibody limbic encephalitis during the same time period

**Case A**	F, 83	6-month history of apathy then drop attacks. Pacemaker inserted. Multiple faciobrachial dystonic seizures.<br><br>MMSE 18/30 Apraxic gait<br><br>Positive Babinski	VGKC-complex	CT brain normal<br><br>EEG slow<br><br>No CSF<br><br>Sodium low	VGKC-complex 8000 pM<br><br>LGI1-Ab strongly positive	VGKC-complex antibody fell to 138 pM with good and sustained response to immunotherapies	Definite autoimmune encephalitis.
**Case B**	M, 83	9 months of short lived episodes of loss of awareness then confusion, apraxic gait, major seizures, akinetic mute.	VGKC-complex	MRI restricted diffusion and swollen left basal ganglia<br><br>EEG slow wave<br><br>CSF 15 WBC<br><br>14.3.3 positive	VGKC-complex 5242 pM.<br><br>LGI1-Ab positive	VGKC-complex confirmed but died before treatment.	PM encephalitis.
**Case C**	M, 71	9 months of faciobrachial dystonic seizures, confusion, ataxia, and	NMDAR, VGKC-complex	MRI vascular disease<br><br>CSF acellular	VGKC-complex 6000 pM,<br><br>LGI1-Ab not	No treatment as died before treatment.	Coroner refused PM.

		hallucinations.<br><br>Eventually akinetic mute.		14.3.3 positive<br><br>Sodium normal	tested		

**Prion protein gene sequencing done in cases 1-6 revealed no mutations.
